# The efficacy of anti-EGFR therapy in treating metastatic colorectal cancer differs between the middle/low rectum and the left-sided colon

**DOI:** 10.1038/s41416-021-01470-2

**Published:** 2021-06-29

**Authors:** Kun-Han Lee, Wei-Shone Chen, Jeng-Kai Jiang, Shung-Haur Yang, Huann-Sheng Wang, Shih-Ching Chang, Yuan-Tzu Lan, Chun-Chi Lin, Hung-Hsin Lin, Sheng-Chieh Huang, Hou-Hsuan Cheng, Yee Chao, Hao-Wei Teng

**Affiliations:** 1 Department of Medical Education, Taipei Veterans General Hospital, Taipei, Taiwan; 2 School of Medicine, National Yang-Ming University, Taipei, Taiwan; 3 School of Medicine, National Yang Ming Chiao Tung University, Hsinchu, Taiwan; 4 Division of Colon and Rectal Surgery, Department of Surgery, Taipei Veterans General Hospital, Taipei, Taiwan; 5 Department of Surgery, National Yang Ming Chiao Tung University Hospital, Yilan, Taiwan; 6 Division of Medical Oncology, Department of Oncology, Taipei Veterans General Hospital, Taipei, Taiwan

**Keywords:** Colorectal cancer, Colorectal cancer, Tumour biomarkers, Colorectal cancer

## Abstract

**Background:**

Clinically, metastatic rectal cancer has been considered a subset of left-sided colon cancer. However, heterogeneity has been proposed to exist between high and middle/low rectal cancers. We aimed to examine the efficacy of anti-epidermal growth factor receptor (EGFR) treatment for middle/low rectal and left-sided colon cancers.

**Methods:**

This study enrolled 609 patients with metastatic colorectal cancer who were treated with anti-EGFR therapy. They were divided into groups based on primary tumour locations: the right-sided colon, the left-sided colon or the middle/low rectum. The efficacy of first-line and non-first-line anti-EGFR treatment was analysed. Genomic differences in colorectal cancer data from The Cancer Genome Atlas (TCGA) were investigated and visualised with OncoPrint and a clustered heatmap.

**Results:**

On first-line anti-EGFR treatment, patients with middle/low rectal tumours had significantly lower progression-free survival, overall survival, and overall response rates (6.8 months, 27.8 months and 43%, respectively) than those with left-sided colon cancer (10.1 months, 38.3 months and 66%, respectively). Similar outcomes were also identified on non-first-line anti-EGFR treatment. In TCGA analysis, rectal tumours displayed genetic heterogeneity and shared features with both left- and right-sided colon cancer.

**Conclusions:**

Anti-EGFR treatment has lower efficacy in metastatic middle/low rectal cancer than in left-sided colon cancer.

## Introduction

Colorectal cancer (CRC) originating from the right and left side of the colon differs in embryologic, epidemiologic, genetic and molecular aspects [1–4]. In the era of targeted therapy, primary tumour location has been found to play an important role in predicting the treatment response and prognosis of metastatic CRC (mCRC). Patients with left-sided mCRC (tumours originating in the splenic flexure, the descending colon, the sigmoid colon, the rectosigmoid junction and sometimes even the rectum) have been demonstrated to have better survival benefits than patients with right-sided mCRC (tumours originating in the caecum, the ascending colon, the hepatic flexure and the transverse colon) [5–7]. Furthermore, the sidedness of primary tumours also determines the efficacy of targeted therapy in treating mCRC, as is the case for anti-epidermal growth factor receptor (EGFR) agents [8–11]. These results were obtained by retrospective analyses of CALGB/SWOG 80405, CRYSTAL and FIRE-3 [12, 13]. In recent years, a comprehensive pooled analysis of six large-scale clinical trials suggested the clear clinical benefit and superior treatment effect of first-line anti-EGFR therapy in patients with *RAS*-wild-type (*RAS*-wt) left-sided mCRC [14].

In terms of mCRC, the rectum has been commonly categorised as part of the left-sided colon in previous trials and research and treated according to the same treatment principles described in the ESMO and NCCN guidelines [15, 16]. However, with the advent of personalised medicine, differences in treatment performance between rectal and left-sided colon tumours have been identified. The left-sided colon and rectum are not merely anatomically and pathologically different [17, 18]. Tumour location (right-sided colon, left-sided colon and rectum) was suggested to serve as a prognostic factor in stage III CRC in a recent study [19]. Furthermore, in patients with mCRC treated with anti-EGFR agents, a higher overall response rate is achieved in left-sided colon tumours than in rectal tumours [20, 21].

The definition of rectal cancer itself, which could differ surgically, anatomically, and/or biologically, remains inconclusive [22, 23]. According to the latest ESMO guidelines, rectal cancer is divided into high, middle and low rectal cancer (>10–15, ≥5–10 and <5 cm from the anal verge, respectively) [15]. Moreover, data have suggested that high rectal tumours have similar characteristics to left-sided CRC, including different lymphatic drainage and vascular supply systems, distinguishing metastatic patterns, response to adjuvant chemotherapy and even survival outcomes [18, 24–28]. Therefore, whether tumours located 10–15 cm from the anal verge should be defined as colon or rectal tumours is still under debate. In clinical practice, during our multidisciplinary team meetings, middle/low rectal cancer was noted to be refractory to anti-EGFR therapy to some extent, and the efficacy of this therapy for middle/low rectal cancer was below expectations compared to left-sided colon cancer. However, there is no current large-scale research investigating this issue.

In our study, we aimed to compare the efficacy of anti-EGFR therapy in treating metastatic middle/low rectal cancers and left-sided colon cancers. Right-sided colon cancers were also added for a complete analysis of the impact of primary tumour locations. CRC data from The Cancer Genome Atlas (TCGA) were analysed to validate our clinical results at a molecular level.

## Methods

### Patients

This is a retrospective large-scale cohort study. Data were collected between May 2005 and December 2019 at the Taipei Veterans General Hospital, Taiwan. Patients with pathologically confirmed mCRC were eligible for enrolment, while those with *RAS* and *BRAF* mutations were excluded due to their unsuitability for anti-EGFR treatment. Patients were classified based on their primary tumour locations, including the right-sided colon (tumours originating in the caecum, ascending colon, hepatic flexure and transverse colon), the left-sided colon (tumours originating in the splenic flexure, descending colon, sigmoid colon, rectosigmoid junction and rectum >10 cm from the anal verge), and the middle/low rectum (≤10 cm from the anal verge). Patients with different primary tumour locations were treated with chemotherapy plus anti-EGFR (cetuximab or panitumumab) or anti-vascular endothelial growth factor (VEGF, bevacizumab) agents. They were divided into two groups based on their treatment sequences: one (the first-line group) receiving anti-EGFR first and the other (the non-first-line group) receiving anti-EGFR following anti-VEGF. All patients were administered at least two cycles of anti-EGFR regimens. The inclusion flowchart is presented in detail in Supplemental Fig. S1. Basic patient clinicopathological information, including age, sex, American Joint Committee on Cancer 7^th^ Edition stage at first presentation (AJCC stage), metastasectomy (surgical resection of metastasis with curative intent during any stage of a patient’s metastatic course), pathology, histological grade, mucinous component, signet cell component, lymphovascular invasion status, perineural invasion status, carcinoembryonic antigen (CEA, in milligrams per decilitre) level and carbohydrate antigen 19-9 (CA199, in milligrams per decilitre) level, was collected. All materials and protocols in this study were compliant with the Declaration of Helsinki and Good Clinical Practice guidelines. The study protocols were approved by the ethics committee and institutional review board of the Taipei Veterans General Hospital.

### Treatment outcomes

Median overall survival (OS) was defined as the time from diagnosis of metastasis to cancer death or loss to follow-up. Median progression-free survival (PFS) represented the time from the beginning of anti-EGFR treatment to disease progression, as confirmed by radiological images (CT, PET/CT, MRI) or intolerable toxicity. The overall response rate (ORR; the proportion of patients with confirmed complete or partial response) and disease control rate (DCR; the proportion of patients with confirmed complete response, partial response or stable disease) of metastatic tumours were recorded based on the Response Evaluation Criteria in Solid Tumours (RECIST) version 1.1 [29]. Patients who achieved partial or complete response were defined as treatment responders, and those who met only the criteria for stable disease or progressive disease were considered non-responders.

### Gene expression analysis

In order to clarify the genomic differences among left-sided colon, right-sided colon and rectal cancers, TCGA colorectal data were utilised as the training set to explore the differential expression of genomic features between left- and right-sided colon cancer. The significantly different expression of genomic features in the training set was compared with the potentially preventive genomic features in rectal cancer as a validation model.

The clinical and molecular information of 594 colorectal tumour samples was acquired from the publicly available “TCGA Pan-Cancer Atlas” project on the cBioPortal TCGA website [30]. Patients with *RAS* or *BRAF* mutations, non-primary tumour samples or unspecified tumour locations were excluded from the evaluation (Supplemental Fig. S2). Samples were then divided into three groups based on their locations according to ICD 10 codes (C18.0, C18.2, C18.3 and C18.4 as right-sided colon, 161 samples; C18.5, C18.6, C18.7 and C19 as left-sided colon, 126 samples; and C20 as rectal cancer, 141 samples). Genomic and epigenomic differences regarding single-nucleotide polymorphisms (SNPs), the transcriptome (mRNA) and DNA methylation were analysed. Aggregated mutation information was obtained in a mutation annotation format, and the number and overlaps of 1–100% SNP mutations between the right-sided colon, left-sided colon and rectal cancer groups were calculated. Differentially methylated region (DMR) detection was utilised to identify possible genetic silencing caused by methylation between right- and left-sided colon tumours. The result was regarded as the control group (training set) and visualised with a volcano plot and a list of heatmaps. The rectal cancer group was then evaluated and compared with the control group. Significance was set to require beta-value differences <0.15 and an adjusted *P* value < 0.05. Differential mRNA expression analysis was conducted with the same method. The percentage differences in mRNA expression in right- and left-sided colon tumours were first collected in the control group. Rectal tumours were then added for comparison. A false discovery rate < 0.01 and log fold change > 1 were set as the cut-off points for significance. All data were processed with TCGAbiolinks and ComplexHeatmap [31–33].

### Statistical analysis

Statistical comparisons were based on nonparametric tests. The correlations between clinicopathological variables and treatment responses were analysed using the chi-square test or Fisher’s exact test, where appropriate. PFS and OS curves were calculated through the Kaplan–Meier method. As for univariable and multivariable analyses, Cox proportional hazards regression was used to determine the effects of primary tumour locations and particular factors and to obtain hazard ratios (HRs) with corresponding two-tailed 95% confidence intervals (CIs). Cox regression models were conducted in the subgroup analysis to evaluate the impact of anti-EGFR treatment in primary tumour locations and sequences stratified by baseline variables. A two-sided *P* value < 0.05 was regarded as statistically significant. All statistical analyses were performed using SPSS 24.0 software (IBM Co., Armonk, NY).

## Results

### Patients

A total of 609 patients were included in this study. The baseline characteristics of patients in the right-sided colon (*n* = 125), the left-sided colon (*n* = 313) and the middle/low rectal (*n* = 171) tumour groups are presented in Table 1. Among them, 39% of right-sided colon, 43% of left-sided colon and 47% of middle/low rectal tumours were treated with first-line anti-EGFR therapy. Several variables, including sex, age, AJCC stage, metastasectomy, histological grade, mucinous component, signet cell component, CEA and CA199 level, led to imbalanced datasets due to the retrospective nature of the study. The characteristics of patients treated with first-line or non-first-line anti-EGFR therapy are listed in Supplemental Tables S1 and S2.

**Table 1:** Baseline characteristics of the patients^a^.

Characteristic	Middle/low rectum (*n* = 171)	Left-sided colon (*n* = 313)	Right-sided colon (*n* = 125)	*P* value
Sex—no. (%)				0.042
Male	107 (63)	203 (65)	65 (52)	
Female	64 (37)	110 (35)	60 (48)	
Age—no. (%)				<0.001
<70 years	139 (81)	239 (76)	78 (62)	
≥70 years	32 (19)	74 (24)	47 (38)	
Line of therapy—no. (%)^b^				0.429
First-line	80 (47)	136 (43)	49 (39)	
Non-first-line	91 (53)	177 (57)	76 (61)	
AJCC stage—no. (%)				<0.001^g^
I	12 (7)	0 (0)	0 (0)	
II	13 (8)	18 (6)	4 (3)	
III	42 (25)	50 (16)	14 (11)	
VI	104 (61)	245 (78)	107 (86)	
Metastasectomy—no. (%)				0.003
Yes	55 (32)	140 (45)	38 (30)	
No	116 (68)	173 (55)	87 (70)	
Pathology—no. (%)				0.256^g^
Adenocarcinoma	163 (95)	304 (97)	117 (94)	
Mucinous adenocarcinoma	7 (4)	8 (3)	8 (6)	
Carcinoma	1 (1)	1 (0)	0 (0)	
Histological grade—no. (%)^c,d^				<0.001
High	19 (13)	11 (4)	25 (23)	
Low	130 (87)	267 (96)	85 (77)	
Mucinous component—no. (%)^c^				0.007
Yes	26 (21)	41 (17)	31 (32)	
No	99 (79)	204 (83)	65 (68)	
Signet cell component—no. (%)^c^				0.005^g^
Yes	6 (5)	4 (2)	9 (9)	
No	118 (95)	240 (98)	89 (91)	
Lymphovascular invasion—no. (%)^c,e^				0.526
Yes	61 (49)	134 (54)	53 (55)	
No	64 (51)	112 (46)	43 (45)	
Perineural invasion—no. (%)^c,e^				0.052
Yes	41 (44)	80 (42)	19 (27)	
No	52 (56)	109 (58)	51 (73)	
Baseline CEA level—no. (%)^c,f^				0.004
<6 mg/dL	63 (40)	76 (26)	44 (37)	
≥6 mg/dL	93 (60)	217 (74)	76 (63)	
Baseline CA199 level—no. (%)^c,f^				0.001
<40 mg/dL	97 (67)	136 (48)	59 (52)	
≥40 mg/dL	48 (33)	145 (52)	55 (48)	

*AJCC* American Joint Committee on Cancer staging system, *CEA* carcinoembryonic antigen, *CA199* carbohydrate antigen 19-9, *EGFR* epidermal growth factor receptor, *VEGF* vascular endothelial growth factor.

^a^Percentages may not add up to exactly 100% due to rounding.

^b^The first-line group received anti-EGFR agents (cetuximab or panitumumab) first, and the non-first-line group received anti-EGFR agents after anti-VEGF agents (bevacizumab).

^c^The sum of the groups within this variable may be not the same as the total number of patients due to incomplete pathology reports or serum biochemistry data for a variety of reasons.

^d^Poorly differentiated or undifferentiated tumours are counted as high-histological-grade tumours. Well-differentiated or moderately differentiated tumours are counted as low histological-grade tumours.

^e^Both intramural and extramural invasions are counted.

^f^The cut-offs for CEA and CA199 are based on the normal reference ranges for cancer surveys at our centre.

^g^*P* value by Fisher’s exact test.

### Efficacy

**Fig. 1:**
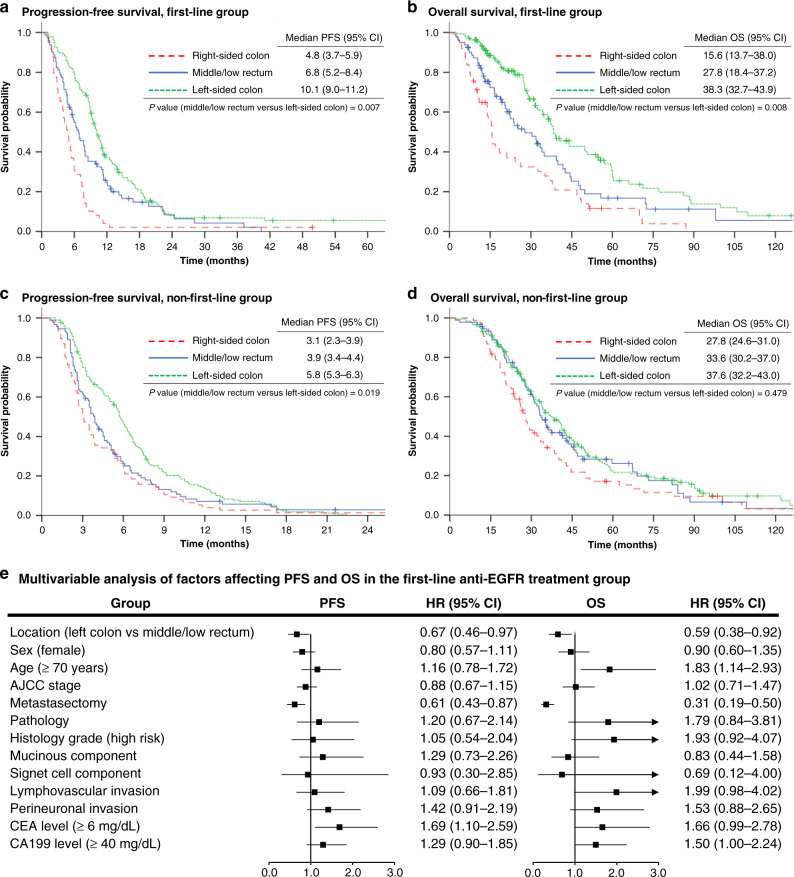
Progression-free survival and overall survival. **a**, **b** represent the progression-free survival (PFS) and overall survival (OS) of the first-line anti-epidermal growth factor receptor (EGFR) treatment group by different primary tumour locations. **c**, **d** represent the PFS and OS of the non-first-line anti-EGFR treatment group by different primary tumour locations. **e** shows the results of multivariate analyses of PFS and OS in patients treated with first-line anti-EGFR therapy. AJCC American Joint Committee on Cancer staging system, CEA carcinoembryonic antigen, CA199 carbohydrate antigen 19-9, HR hazard ratio, CI confidence interval.

**Fig. 2:**
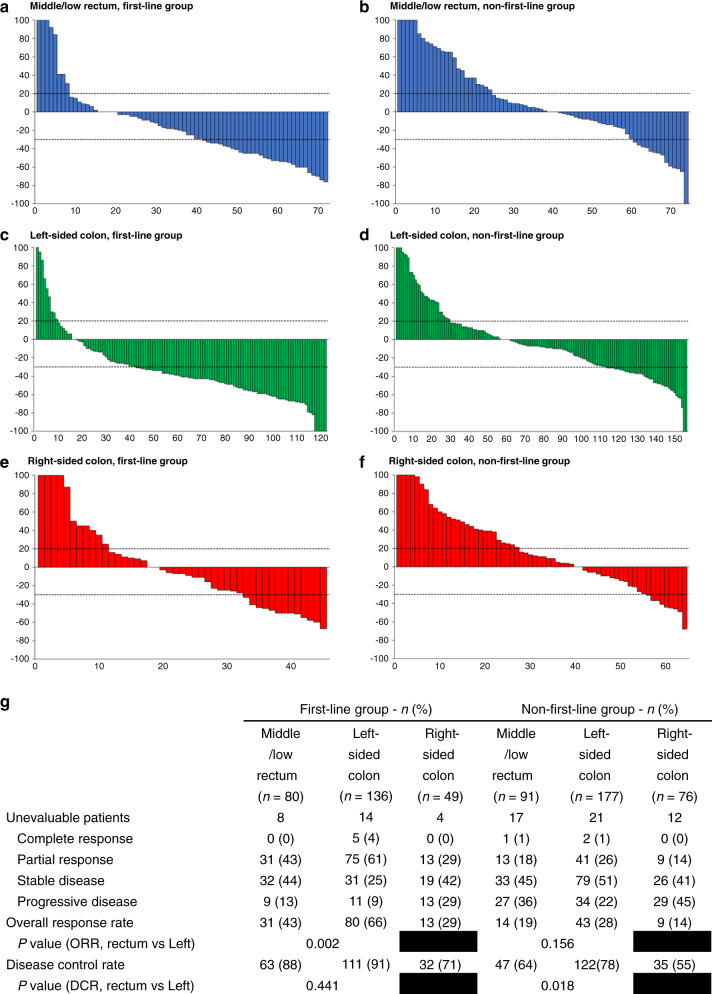
Best percentage change in the size of metastasis and best response in patients. **a**–**f** show the waterfall plots of the best percentage change in the size of target lesions in each patient from the six groups. The analysis is based on Response Evaluation Criteria in Solid Tumours, version 1.1 (RECIST 1.1). The dashed lines at 20% and −30% represent progressive disease and partial response. The line at 0% means either no changes in metastatic sites or the development of a new lesion. **g** shows the tumour response in patients in different groups and is also evaluated based on RECIST 1.1. *n* number, ORR overall response rate, DCR disease control rate.

The PFS, OS, ORR and DCR results from different groups based on primary tumour location and lines of anti-EGFR treatment are presented in Figs. 1 and 2, with waterfall plots showing the best percentage change in metastatic lesions from baseline.

In terms of the first-line anti-EGFR therapy group, PFS (Fig. 1a: 10.1 months vs 6.8 months, HR: 0.67, 95% CI: 0.46–0.89, *P* = 0.007), OS (Fig. 1b: 38.3 months vs 27.8 months, HR: 0.62, 95% CI: 0.44–0.88, *P* = 0.008), and ORR (Fig. 2g: 66% vs 43%, *P* = 0.002) were significantly higher in the left-sided CRC group than in the middle/low rectal tumour group. Significantly longer PFS (Fig. 1c: 5.8 months vs 3.9 months, HR: 0.73, 95% CI: 0.56–0.95, *P* = 0.019) and DCR (Fig. 2g: 78% vs 64%, *P* = 0.018) were also observed in patients with left-sided tumours compared to middle/low rectal tumours under non-first-line anti-EGFR therapy. Conversely, a comparable OS outcome was observed between left-sided tumours and middle/low rectal tumours (Fig. 1d: 37.6 months vs 33.6 months, HR: 0.90, 95% CI: 0.68–1.20, *P* = 0.479) under non-first-line anti-EGFR therapy. The group of patients with right-sided CRC showed the worst PFS, OS, ORR and DCR in both first-line and non-first-line anti-EGFR treatment. The characteristics of treatment responders and non-responders among middle/low rectal cancer patients are summarised in Supplemental Table S3.

Further analysis of the traditionally defined rectal cancer group (with a cut-off of 15 cm from the anal verge) revealed no significant differences in PFS (8.0 months vs 9.3 months, *P* = 0.136) and DCR (90% vs 90%, *P* = 0.949) between the left-sided colon and rectum groups under first-line anti-EGFR treatment (Supplemental Tables S4 and S5).

### Factors affecting PFS and OS in mCRC

**Fig. 3:**
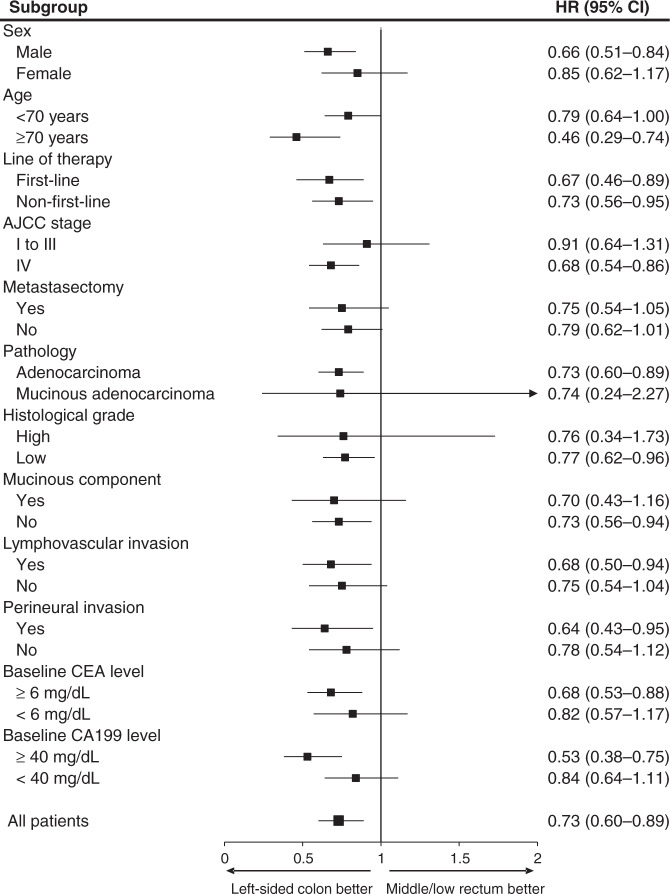
Forest plot of progression-free survival for anti-EGFR treatment recipients with left-sided colon versus middle/low rectal tumours among different subgroups. EGFR epidermal growth factor receptor, AJCC American Joint Committee on Cancer staging system, CEA carcinoembryonic antigen, CA199 carbohydrate antigen 19-9, HR hazard ratio, CI confidence interval.

In order to classify the prognostic role of the primary tumour locations in patients with anti-EGFR treatment, univariate and multivariate analyses were performed (Fig. 1e and Supplemental Tables S6, S7, S8). After adjustment for confounding factors, the middle/low rectal tumour group was identified as an independent factor associated with worse treatment response and prognosis, including shorter PFS and OS under first-line therapy and shorter PFS under non-first-line therapy. In several subgroups, including the subgroups defined by male sex, age ≥70 years, AJCC stage IV, adenocarcinoma pathology, low histological grades, lack of a mucinous component, presence of lymphovascular or perineural invasion, and CEA ≥ 6 mg/dL or CA199 ≥ 40 mg/dL, patients with left-sided colon cancer had better progression-free survival than the equivalent middle/low rectal tumour subgroups (Fig. 3).

### OS according to primary tumour locations and treatment sequences in mCRC patients

Subgroup analysis of OS based on primary tumour locations and sequences of anti-EGFR treatment was also performed in all mCRC patients and the subpopulation of middle/low rectal cancer patients. For both groups, patients without metastasectomy showed superior prognostic results when treated with non-first-line therapy (Supplemental Figs. S3 and S4). Clinical details on patients undergoing metastasectomy in general and hepatic metastasectomy, in particular, are listed in Supplemental Tables S9 and S10.

### Epigenomic and genomic difference analysis

**Fig. 4:**
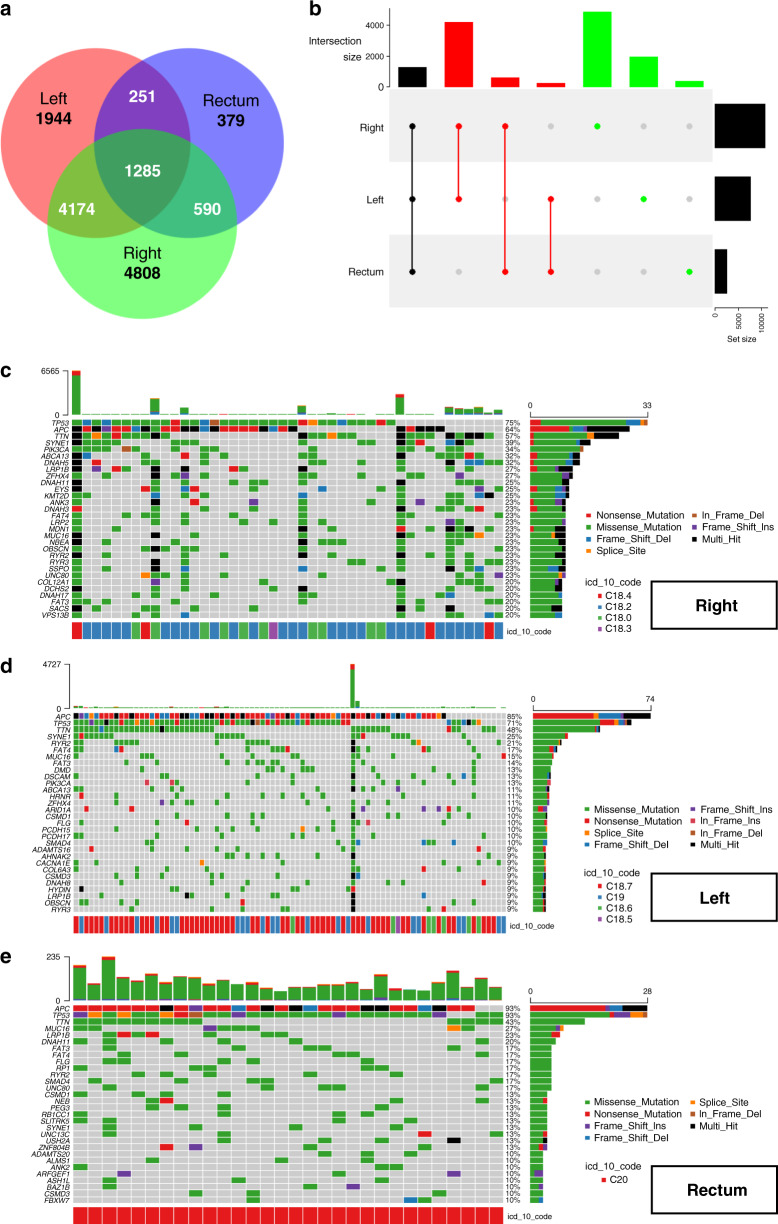
Overlaps and details of SNP mutations among different tumour locations. A Venn diagram (**a**) and an UpSet plot (**b**) show the number of overlaps among different primary tumour locations. **c**–**e** represent the OncoPrints of the right-sided colon, left-sided colon, and rectal tumours, respectively. The top 30 mutated genes and their mutational types and percentages are visualised in detail.

Genetic bioinformation in the mutation annotation format from 161 tumour samples with 1–100% SNP mutations was grouped by primary tumour location and visualised. The Venn diagram (Fig. 4a) and UpSet plot (Fig. 4b) revealed that right-sided colon tumours (*n* = 4808) had more SNP mutations than left-sided colon (*n* = 1944) or rectal (*n* = 379) tumours. There were more SNP overlaps between rectal and right-sided colon tumours (*n* = 590) than between rectal and left-sided colon tumours (*n* = 251). The genomic distribution and types of SNPs based on tumour locations are detailed in Fig. 4c–e. The top 30 most frequently mutated genes, along with their frequencies and mutation types, are reported in the columns.

**Fig. 5:**
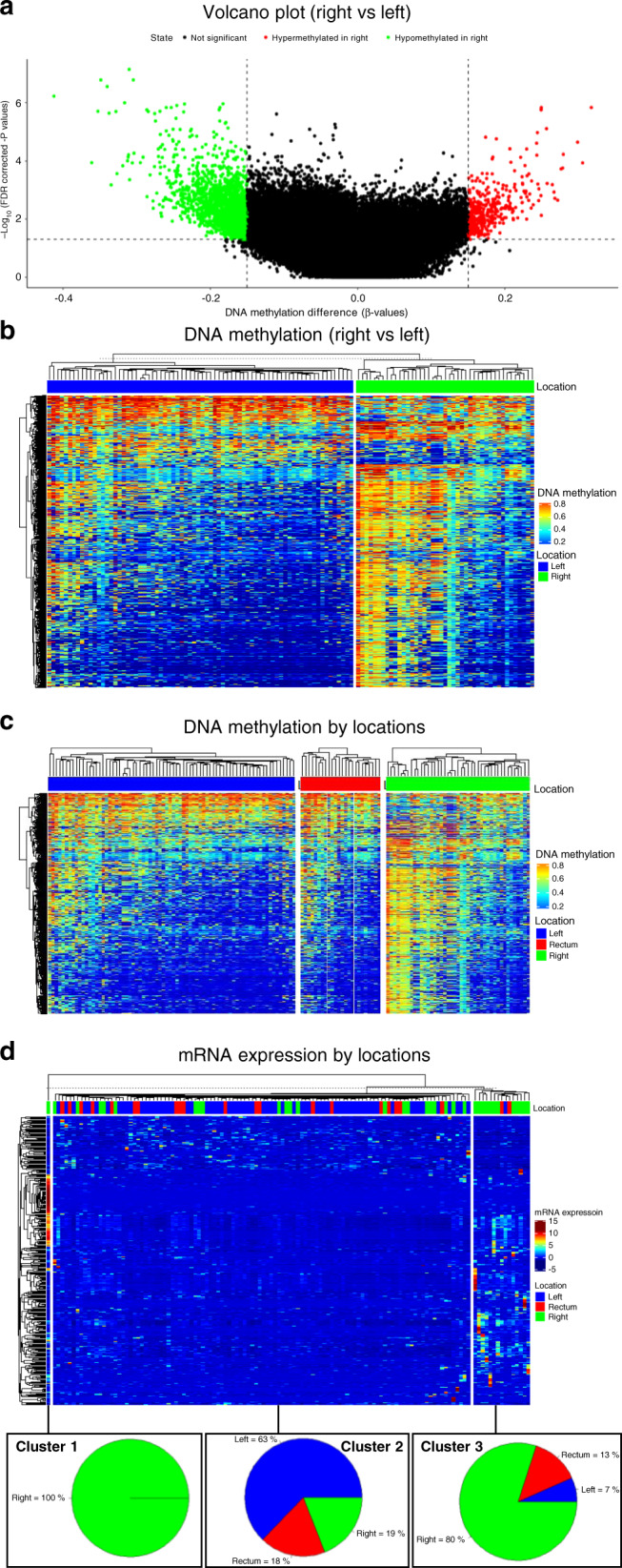
Differences in DNA methylation and mRNA expression among different tumour locations. A volcano plot (**a**) and a list of heatmaps (**b**) were used to demonstrate the DMR between the right and left-sided colon. A list of heatmaps (**c**) was made after adding rectal tumours for comparison. A clustered heatmap (**d**) of mRNA expression differences was generated by *k*-means clustering (*k* = 3) based on the above steps.

For the epigenomic DNA methylation analysis, a volcano plot (Fig. 5a) and a list of heatmaps (Fig. 5b) revealed the DMRs among right- and left-sided colon tumours. Generally, differences in hyper- and hypomethylation of genes were easily observed between right- and left-sided colon cancer. Rectal cancer was introduced into the analysis, and its similarities and differences with right- and left-sided colon cancer are described in Fig. 5c and Supplemental Fig. S5. The same method of mRNA expression analysis was also performed and visualised in Supplemental Figs. S6 and S7. Comparisons across the three tumour groups are illustrated as a clustered heatmap (Fig. 5d) or a list of heatmaps (Supplemental Fig. S8). The rectal cancer group revealed a heterogeneous distribution, showing similarities in both DMR and differential mRNA expression with the left- and right-sided colon cancer groups. Other SNP, DMR and mRNA epigenomic and genomic details are listed in the Dataset file.

## Discussion

Primary tumour location has been considered a useful biomarker to predict the prognosis and the treatment effects of bioagents in mCRC. Although the rectum is routinely grouped in the left-sided colon in current treatment guidelines, this large-scale retrospective study, nevertheless, demonstrates that anti-EGFR therapy has lower efficacy in metastatic middle/low rectal cancer than in left-sided colon cancer, potentially leading to better clinical practice principles for mCRC management.

In our TCGA investigation, distinctions in a genomic level between the rectal and the left-sided colon tumours are identified. The molecular differences serve some possible explanations to our clinical findings. In Fig. 4a, b, the Venn diagram and UpSet plot demonstrate that rectal tumours have more SNP overlaps with right-sided than with left-sided colon tumours. In addition, the OncoPrint in Fig. 4c–e reveals that right-sided colon tumours (20–75%, median = 23%) have the highest mutation rates in their top 30 mutated genes, followed by rectal (10–93%, median = 13%) and left-sided colon (9–85%, median = 10%) tumours. These results indicate that the rectum is not substantially identical to the left-sided colon. From the following analyses of epigenetic DNA methylation and mRNA expression, a list of heatmaps and a clustered heatmap (*k*-means clustering, *k* = 3) are presented in Fig. 5c, d. Rectal cancer showed features resembling those of both left- and right-sided colon cancers. To our knowledge, the growing evidence supports the notion that genomic heterogeneity exists between the colon and the rectal tumours. For example, increased TOPO1 and ERCC1 expression, HER2/neu amplification, and *KRAS* mutation rates in rectal cancer compared with right- and left-sided colon cancer were reported [34, 35]. Lee et al. also described a linear tendency of the decreased mutation incidence for the TGF-β, PI3K and RTK-RAS pathways from the right-sided colon to rectal tumours [36].

Clinically, the differential efficacy of anti-EGFR therapy between the rectal and the left-sided colon tumours was proposed in the previous literature. The analysis of a Phase III trial of panitumumab (PICCOLO) reported similar PFS outcomes between rectal and right-sided colon cancers in *RAS*-wt mCRC patients and mentioned that the benefit of anti-EGFR agents against rectal cancers may be overestimated [37]. Loupakis et al. observed a reduced OS (19.6 vs 22.8 months, *P* = 0.028) and ORR (35.9% vs 48.9%, *P* = 0.019) of rectal tumours in the AVF2107g trial [20]. Another retrospective analysis of two Phase II Spanish trials described a reduced unconfirmed ORR (64% vs 80%, CI = 0.2–0.9) but similar PFS and OS in the rectal tumour group treated with anti-EGFR [21], which is different from our results to some degree. The main reason might be that different definitions of rectal cancer were used. Although the traditional classification of rectal cancer is within 15 cm of the anal verge, emerging studies regard the high rectum as a part of the left-sided colon in terms of pathological, physiological and clinical outcomes. A meta-analysis including 1196 patients with stage II or III rectal cancer suggested that tumours located within 10–15 cm from the anal verge might benefit from adjuvant chemotherapy, with higher disease-free survival and lower distant recurrence rate than middle/low rectal tumours [24]. Several retrospective studies also reported that OS and disease-free survival in high rectal cancer were similar to those in sigmoid colon cancer but superior to those in middle/low rectal cancer in non-metastatic CRC without adjuvant therapy [18, 26, 28]. Hence, in this study, tumours within 10 cm from the anal verge were assigned to the middle/low rectal tumour group, while those within 10–15 cm were assigned to the left-sided colon group. Our findings (Figs. 1 and 2) supported the assumption of worse PFS, OS and ORR among the middle/low rectal tumour group than among the left-sided colon group. The negative prognostic impact of middle/low rectal cancer in patients treated with first-line anti-EGFR was found to be independent in multivariate analyses of PFS and OS (Fig. 1e). However, regarding the clinical features of responding and non-responding middle/low rectal cancer patients under first-line anti-EGFR treatment, no significant differences were found (Supplemental Table S3).

Analyses of left-sided colon and rectal tumours were also performed using the traditional cut-off point (15 cm from the anal verge) with the aim of testing our hypothesis (Supplemental Tables S4 and S5). The results were similar to those reported by Loupakis et al. and Benavides et al., whereby rectal tumours were associated specifically with worse OS (32.1 months vs 38.3 months, *P* = 0.010) and ORR (49% vs 64%, *P* = 0.035) than left-sided colon tumours, while no significant differences in PFS (8.0 months vs 9.3 months, *P* = 0.136) and DCR (90% vs 90%, *P* = 0.949) were seen in the first-line anti-EGFR treatment group [20, 21]. Meanwhile, there were no significant differences in any of those variables in the non-first-line treatment group.

In several previous studies, anti-EGFR as a first-line treatment has shown higher efficacy and a greater survival benefit than anti-VEGF in treating left-sided, *RAS*-wt and mCRC patients [14]. However, there was a trend toward shorter OS in middle/low rectal cancer patients who received anti-EGFR treatment as the first line than in those who received non-first-line EGFR treatment (27.8 months vs 33.6 months, *P* = 0.124). Thus, the guiding principles for mCRC treatment selection according to primary tumour location, especially tumours in the middle/low rectum, need further evaluation and confirmation.

Subgroup analyses for all patients (Supplemental Fig. S3) and the limited population of patients with middle/low rectal cancer (Supplemental Fig. S4) indicate that patients without metastasectomy showed higher OS in the non-first-line anti-EGFR treatment group than in the first-line group. Metastasectomy has been proposed to benefit patients with mCRC, and anti-EGFR therapy was reported to produce an improved ORR and increased resectability of metastatic lesions [38–42]. However, in our study, a reduced ORR was observed in middle/low rectal cancer treated with anti-EGFR therapy, potentially leading to the unsatisfactory OS in first-line anti-EGFR treatment. We noted with interest that a recently published article from our centre described a 32% reduction in the death risk in the left-sided colon compared with right-sided colon cancer when hepatic metastasectomy was performed on patients with colorectal liver metastasis, whereas no OS difference was noted between the rectal and right-sided colon cancer groups [43]. The correlation between metastasectomy and the nature of primary tumour locations is worth investigating when targeted agents become an important confounding factor in the prognostic results.

This study was limited by its single-centre and retrospective nature. Some pathological data were not recorded due to the unresectable condition of the primary tumours. In addition, control arms consisting of mCRC patients treated with anti-VEGF therapy or chemotherapy only were not included in this study. The particular subgroup of interest in this study, namely, patients with middle/low rectal cancers, had only a modest sample size available for analysis, and the results need to be validated by future studies. The impact of bioagent treatment sequencing and efficacy by primary tumour location requires further investigation in the form of randomised controlled trials or head-to-head comparisons. Finally, we have evaluated only the bioinformatic differences among right-sided colon and left-sided colon cancer and rectal cancer. The molecular characteristics of different parts of the rectum (high vs middle/low) remain to be investigated. Specific genomic features affecting the efficacy and resistance rate of anti-EGFR therapy should also be explored to demonstrate their relationship with primary tumour locations.

In conclusion, the differential efficacy of anti-EGFR treatment between the middle/low rectal cancers and the left-sided colon cancers has been established. Moreover, TCGA genomic analysis partially supported our findings that rectal cancer is not a homogeneous group and that it shares features with both left-sided colon and right-sided colon cancer. The usefulness of primary tumour location for the basis of anti-EGFR treatment decisions warrants further exploration.

## Supplementary information

Supplemental information

Dataset 1

## Data Availability

The datasets generated and/or analysed during this study are available from the corresponding author on reasonable request.
