# Supplementary material for: The efficacy of anti-EGFR therapy in treating metastatic colorectal cancer differs between the middle/low rectum and the left-sided colon
Source: Br J Cancer. 2021 Jun 29;125(6):816–25. doi: 10.1038/s41416-021-01470-2 (PMC8437976; doi:10.1038/s41416-021-01470-2)
Supplement: Supplementary file 1 — Supplemental information [file 41416_2021_1470_MOESM1_ESM.docx]

**Supplemental information**

**The efficacy of anti-EGFR therapy in treating metastatic colorectal cancer differs between the middle/low rectum and the left-sided colon**

**Kun-Han Lee, Wei-Shone Chen, Jeng-Kai Jiang, Shung-Haur Yang, Huann-Sheng Wang, Shih-Ching Chang, Yuan-Tzu Lan, Chun-Chi Lin, Hung-Hsin Lin, Sheng-Chieh Huang, Hou-Hsuan Cheng, Yee Chao, Hao-Wei Teng**

**Supplemental Figure S1. Enrolment flowchart for anti-EGFR treatment with different primary tumour locations**


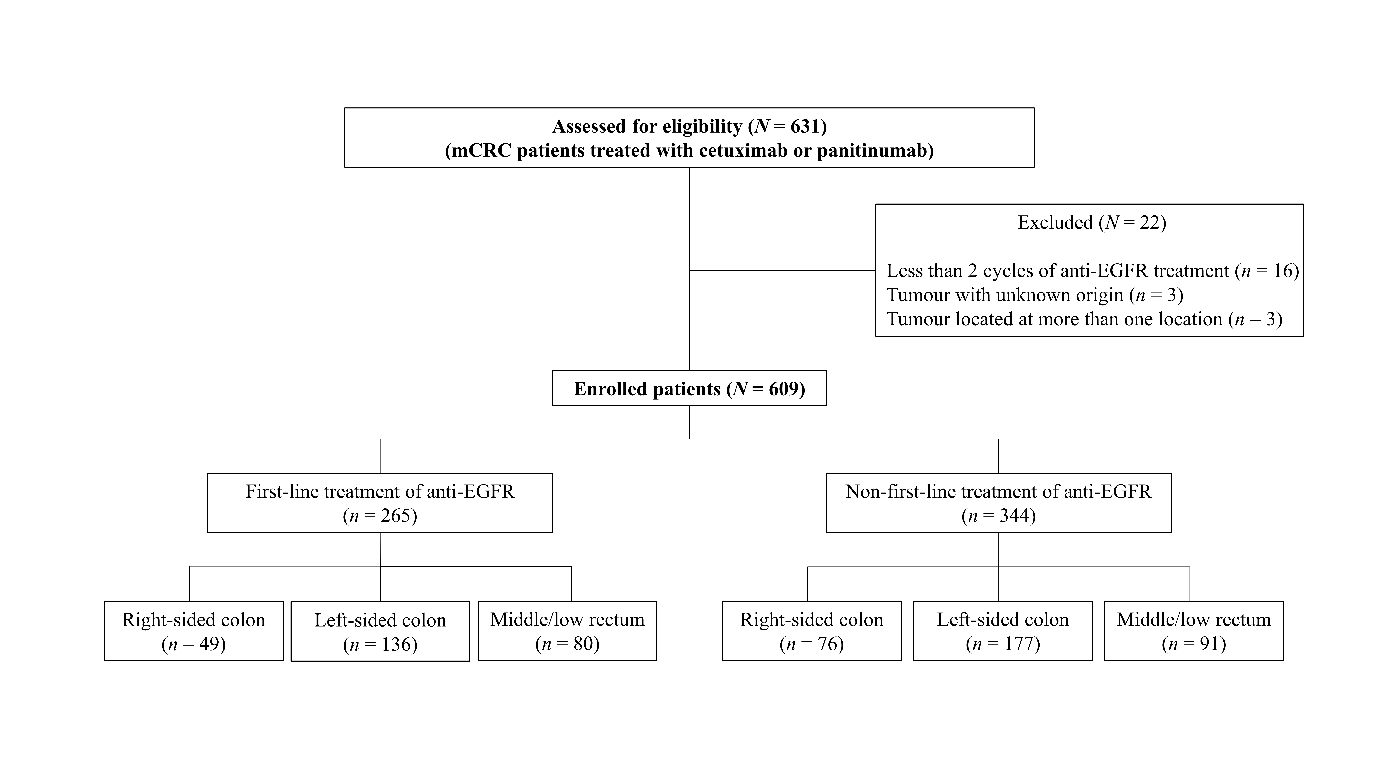


Abbreviations: EGFR, epidermal growth factor receptor; mCRC, metastatic colorectal cancer

**Supplemental Figure S2. Enrolment flowchart for genomic bioinformatic analysis**


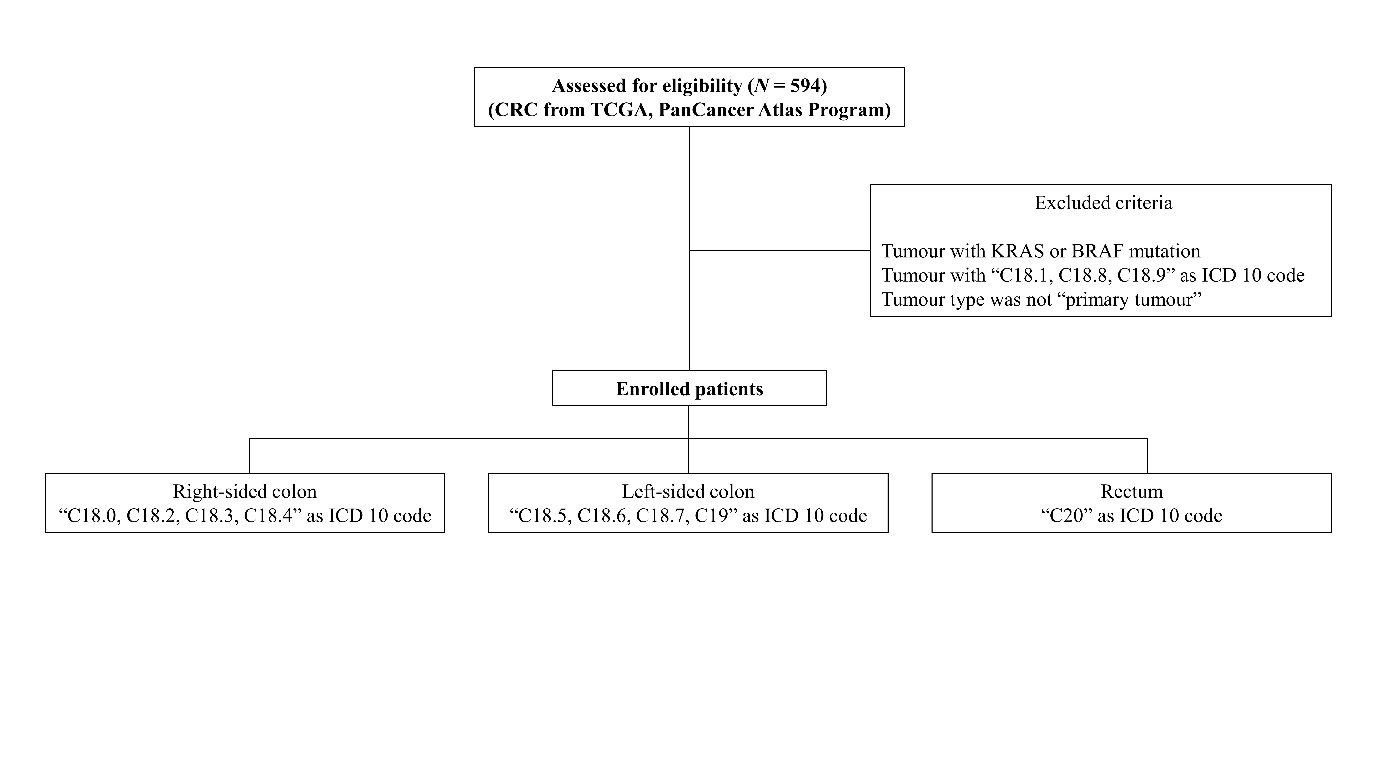


Abbreviations: CRC, colorectal cancer; TCGA, The Cancer Genome Atlas

**Supplemental Figure S3. Forest plot of overall survival for mCRC patients treated with first-line versus non-first-line anti-EGFR treatment across different subgroups**

First-line Better

Non-first-line Better

Abbreviations: EGFR, epidermal growth factor receptor; AJCC, American Joint Committee on Cancer**;** CEA, carcinoembryonic antigen**;** CA199, carbohydrate antigen 19-9; HR, hazard ratio; CI, confidence interval

**Supplemental Figure S4. Forest plot of overall survival for patients with middle/low rectal tumours treated with first-line versus non-first-line anti-EGFR treatment across different subgroups**

Non-first-line Better

First-line Better

Abbreviations: EGFR, epidermal growth factor receptor; AJCC, American Joint Committee on Cancer**;** CEA, carcinoembryonic antigen**;** CA199, carbohydrate antigen 19-9; HR, hazard ratio; CI, confidence interval

**Supplemental Figure S5. A clustered heatmap (*k* = 3 for *k*-means clustering) of DNA methylation among different primary tumour locations**


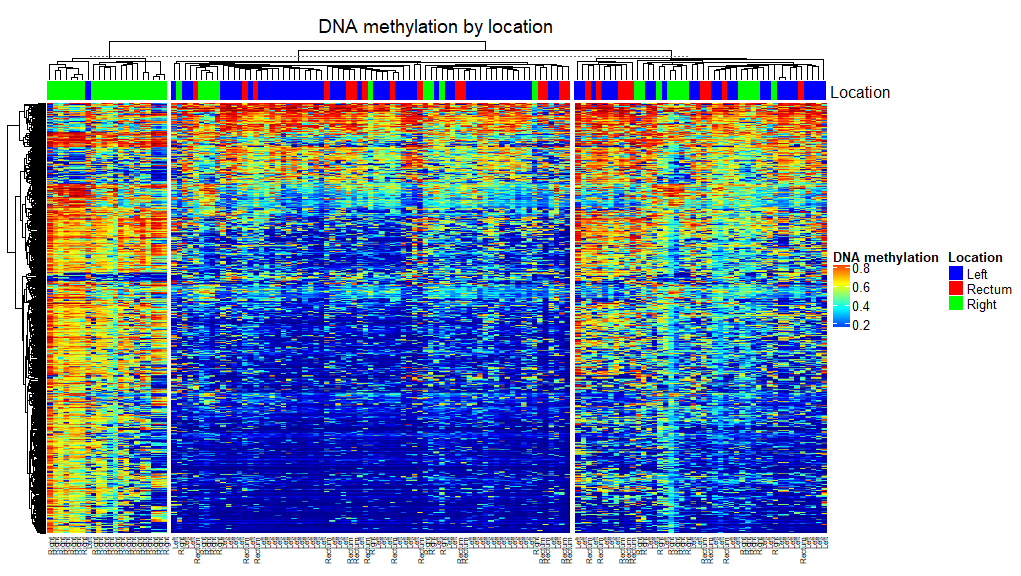

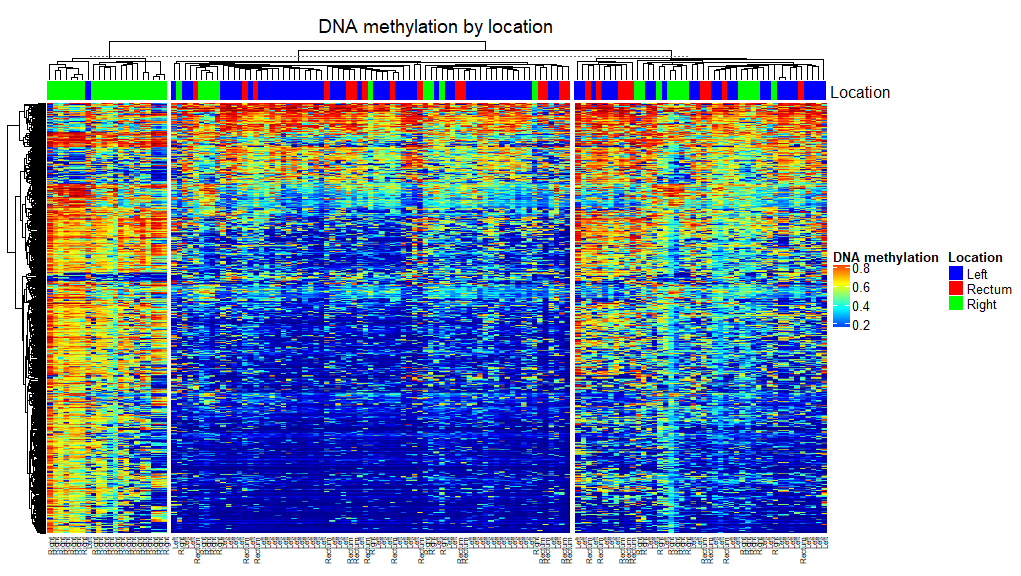


**Supplemental Figure S6. Volcano plot for differential expression analysis of mRNA between right- and left-sided colon tumours**


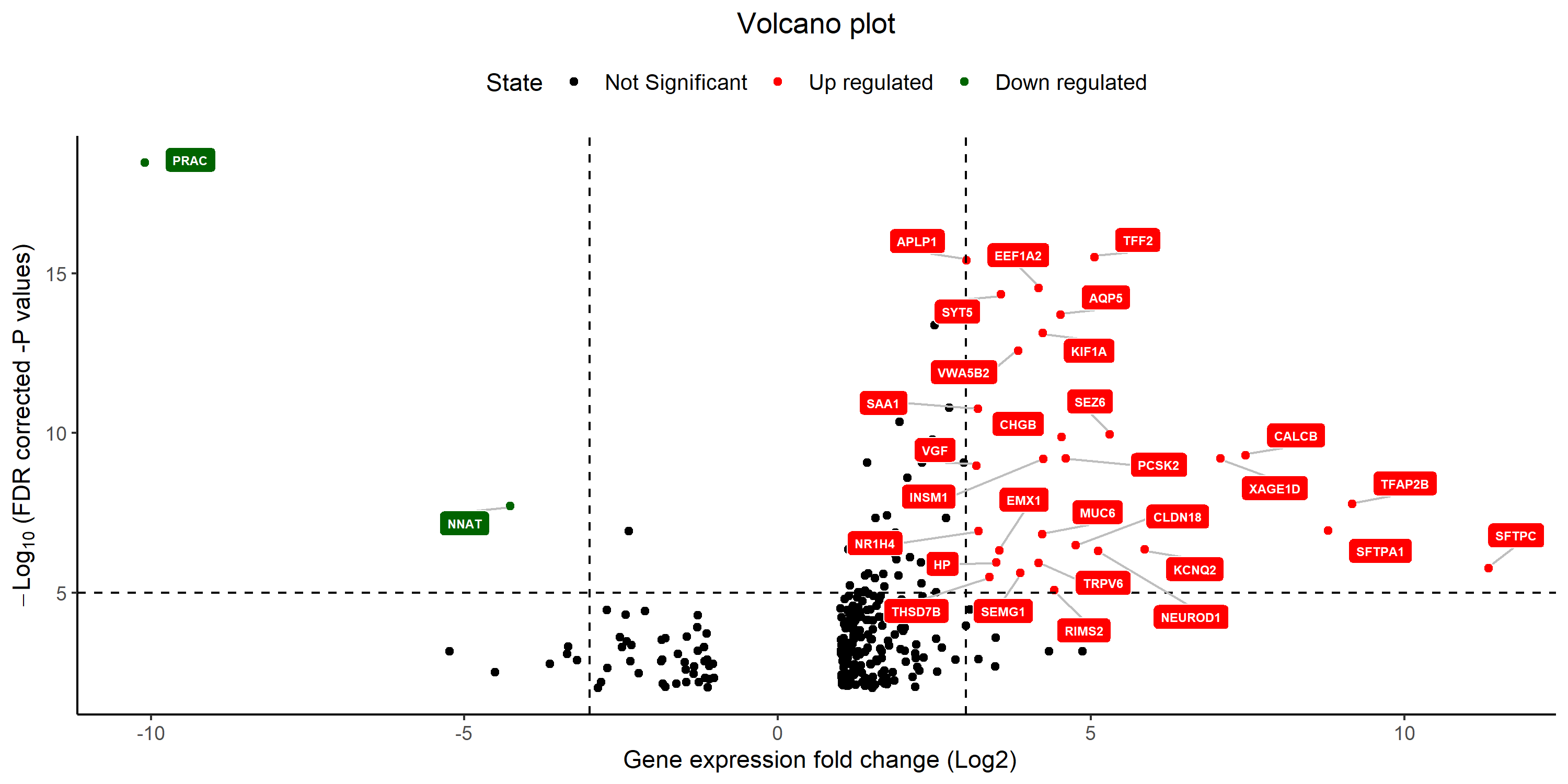


**Supplemental Figure S7. A list of heatmaps of mRNA-level gene expression in mRNA between right- and left-sided colon tumours**


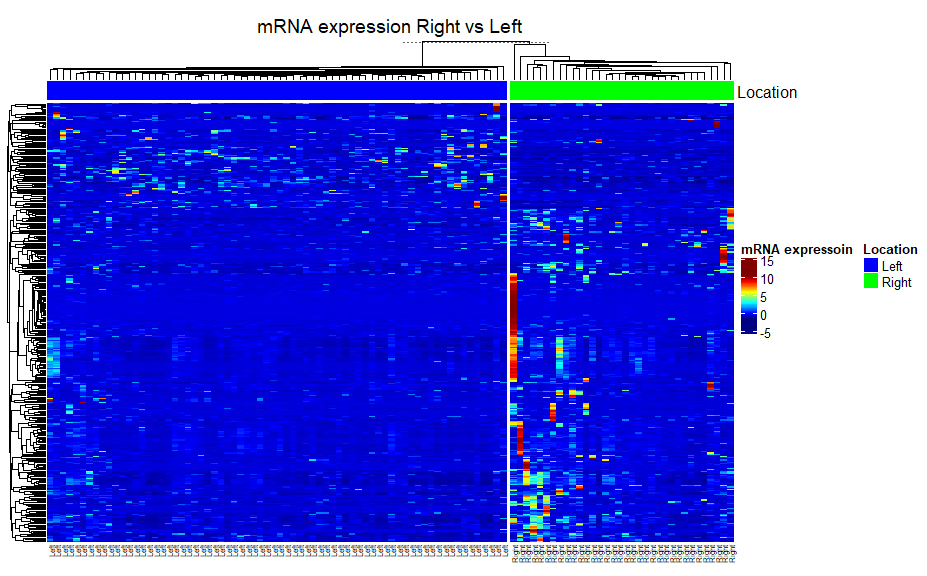

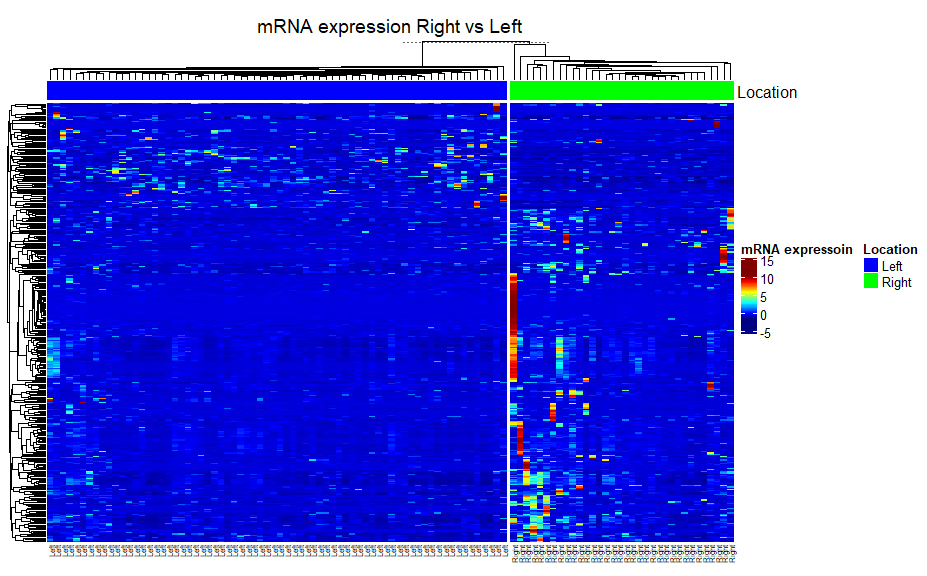


**Supplemental Figure S8. A list of heatmaps of gene expression in mRNA among different primary tumour locations**


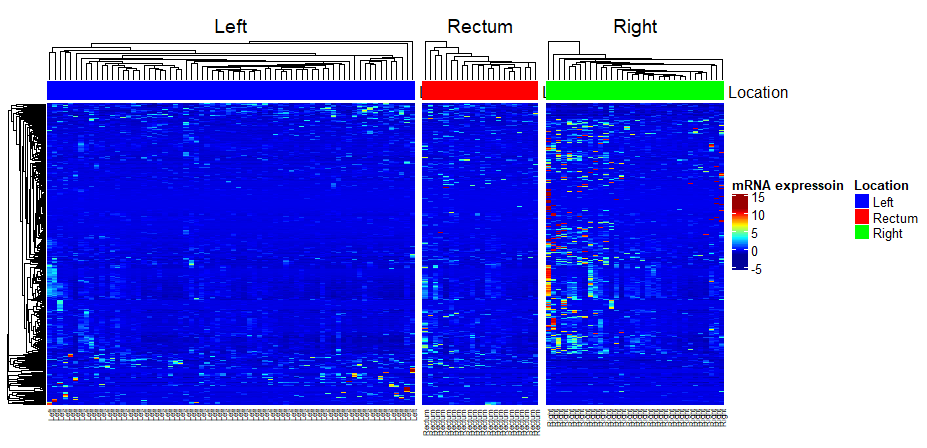

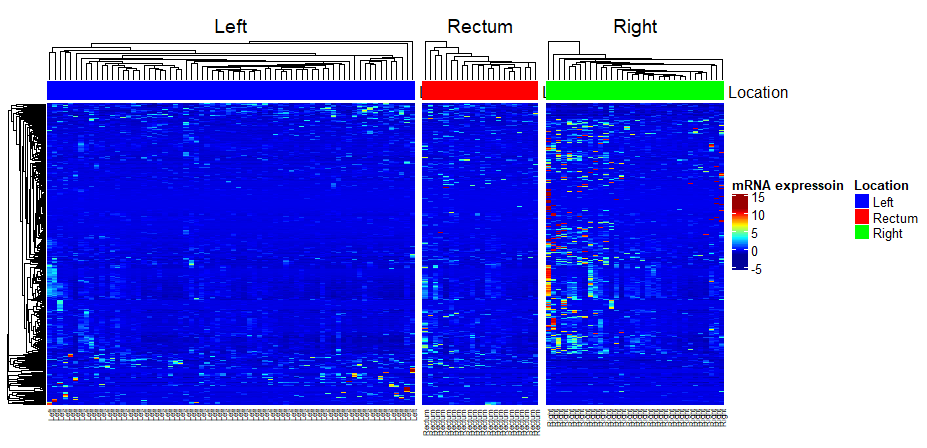


| \| **Supplemental Table S1. Baseline characteristics of patients who received first-line anti-EGFR treatment** \| \| \| \| \| \| --- \| --- \| --- \| --- \| --- \| \| **Characteristic – no. (%)** \| **Middle/low rectum (*n* = 80)** \| **Left-sided colon  (*n* = 136)** \| **Right-sided colon (*n* = 49)** \| ***P*-value** \| \| Sex – no. (%) \| \| \| \| 0.015 \| \| Male \| 52 (65) \| 89 (65) \| 21 (43) \|  \| \| Female \| 28 (35) \| 47 (35) \| 28 (57) \|  \| \| Age – no. (%) \| \| \| \| 0.104 \| \| < 70 years \| 64 (80) \| 102 (75) \| 31 (63) \|  \| \| ≥ 70 years \| 16 (20) \| 34 (25) \| 18 (37) \|  \| \| AJCC stage – no. (%) \| \| \| \| <0.001^a^ \| \| I \| 7 (9) \| 0 (0) \| 0 (0) \|  \| \| II \| 2 (3) \| 6 (4) \| 0 (0) \|  \| \| III \| 17 (21) \| 11 (8) \| 3 (6) \|  \| \| VI \| 54 (68) \| 119 (88) \| 46 (94) \|  \| \| Metastasectomy – no. (%) \| \| \| \| <0.001 \| \| Yes \| 25 (31) \| 67 (49) \| 10 (20) \|  \| \| No \| 55 (69) \| 69 (51) \| 39 (80) \|  \| \| Pathology – no. (%) \| \| \| \| 0.035^a^ \| \| Adenocarcinoma \| 78 (98) \| 133 (98) \| 44 (90) \|  \| \| Mucinous adenocarcinoma \| 2 (3) \| 2 (1) \| 5 (10) \|  \| \| Carcinoma \| 0 (0) \| 1 (1) \| 0 (0) \|  \| \| Histological grade – no. (%) \| \| \| \| 0.097 \| \| High \| 11 (15) \| 8 (7) \| 6 (15) \|  \| \| Low \| 60 (85) \| 113 (93) \| 33 (85) \|  \| \| Mucinous component – no. (%) \| \| \| \| 0.013 \| \| Yes \| 11 (20) \| 12 (11) \| 11 (32) \|  \| \| No \| 43 (80) \| 96 (89) \| 23 (68) \|  \| \| Signet cell component – no. (%) \| \| \| \| 0.086^a^ \| \| Yes \| 3 (6) \| 1 (1) \| 2 (6) \|  \| \| No \| 51 (94) \| 107 (99) \| 32 (94) \|  \| \| Lymphovascular invasion – no. (%) \| \| \| \| 0.878 \| \| Yes \| 32 (58) \| 63 (57) \| 18 (53) \|  \| \| No \| 23 (42) \| 47 (43) \| 16 (47) \|  \| \| Perineural invasion – no. (%) \| \| \| \| 0.006^a^ \| \| Yes \| 21 (50) \| 39 (46) \| 3 (13) \|  \| \| No \| 21 (50) \| 46 (54) \| 20 (87) \|  \| \| Baseline CEA level – no. (%) \| \| \| \| 0.016 \| \| < 6 mg/dL \| 29 (39) \| 27 (20) \| 12 (24) \|  \| \| ≥ 6 mg/dL \| 46 (61) \| 105 (80) \| 37 (76) \|  \| \| Baseline CA199 level – no. (%) \| \| \| \| 0.004 \| \| < 40 mg/dL \| 49 (66) \| 55 (43) \| 21 (45) \|  \| \| ≥ 40 mg/dL \| 25 (34) \| 74 (57) \| 26 (55) \|  \| |
| --- | --- | --- | --- | --- | --- | --- | --- | --- | --- | --- | --- | --- | --- | --- | --- | --- | --- | --- | --- | --- | --- | --- | --- | --- | --- | --- | --- | --- | --- | --- | --- | --- | --- | --- | --- | --- | --- | --- | --- | --- | --- | --- | --- | --- | --- | --- | --- | --- | --- | --- | --- | --- | --- | --- | --- | --- | --- | --- | --- | --- | --- | --- | --- | --- | --- | --- | --- | --- | --- | --- | --- | --- | --- | --- | --- | --- | --- | --- | --- | --- | --- | --- | --- | --- | --- | --- | --- | --- | --- | --- | --- | --- | --- | --- | --- | --- | --- | --- | --- | --- | --- | --- | --- | --- | --- | --- | --- | --- | --- | --- | --- | --- | --- | --- | --- | --- | --- | --- | --- | --- | --- | --- | --- | --- | --- | --- | --- | --- | --- | --- | --- | --- | --- | --- | --- | --- | --- | --- | --- | --- | --- | --- | --- | --- | --- | --- | --- | --- | --- | --- | --- | --- | --- | --- | --- | --- | --- | --- | --- | --- | --- | --- | --- | --- | --- | --- | --- | --- | --- | --- | --- | --- | --- | --- | --- | --- | --- | --- | --- | --- | --- | --- | --- | --- | --- | --- | --- | --- | --- | --- | --- | --- | --- | --- | --- | --- | --- | --- | --- | --- | --- | --- | --- | --- | --- |

^a^ *P*-value by Fisher’s exact test

Abbreviations: EGFR, epidermal growth factor receptor; AJCC, American Joint Committee on Cancer**;** CEA, carcinoembryonic antigen**;** CA199, carbohydrate antigen 19-9

| **Supplemental Table S2. Baseline characteristics of patients who received non-first-line anti-EGFR treatment** | | | | |
| --- | --- | --- | --- | --- |
| **Characteristic – no. (%)** | **Middle/low rectum (*n* = 91)** | **Left-sided colon  (*n* = 177)** | **Right-sided colon (*n* = 76)** | ***P*-value** |
| Sex – no. (%) | | | | 0.585 |
| Male | 55 (60) | 114 (64) | 44 (58) |  |
| Female | 36 (40) | 63 (36) | 32 (42) |  |
| Age – no. (%) | | | | 0.006 |
| < 70 years | 75 (82) | 137 (77) | 47 (62) |  |
| ≥ 70 years | 16 (18) | 40 (23) | 29 (38) |  |
| AJCC stage – no. (%) | | | | 0.001^a^ |
| I | 5 (5) | 0 (0) | 0 (0) |  |
| II | 11 (12) | 12 (7) | 4 (5) |  |
| III | 25 (27) | 39 (22) | 11 (14) |  |
| VI | 50 (55) | 126 (71) | 61 (80) |  |
| Metastasectomy – no. (%) | | | | 0.405 |
| Yes | 30 (33) | 73 (41) | 28 (37) |  |
| No | 61 (67) | 104 (59) | 48 (63) |  |
| Pathology – no. (%) | | | | 0.513^a^ |
| Adenocarcinoma | 85 (93) | 171 (97) | 73 (96) |  |
| Mucinous adenocarcinoma | 5 (5) | 6 (3) | 3 (4) |  |
| Carcinoma | 1 (1) | 0 (0) | 0 (0) |  |
| Histological grade – no. (%) | | | | <0.001^a^ |
| High | 8 (10) | 3 (2) | 19 (27) |  |
| Low | 70 (90) | 154 (98) | 52 (73) |  |
| Mucinous component – no. (%) | | | | 0.196 |
| Yes | 15 (21) | 29 (21) | 20 (32) |  |
| No | 56 (79) | 108 (79) | 42 (68) |  |
| Signet cell component – no. (%) | | | | 0.030^a^ |
| Yes | 3 (4) | 3 (2) | 7 (11) |  |
| No | 67 (96) | 133 (98) | 57 (89) |  |
| Lymphovascular invasion – no. (%) | | | | 0.188 |
| Yes | 29 (41) | 71 (52) | 35 (56) |  |
| No | 41 (59) | 65 (48) | 27 (44) |  |
| Perineural invasion – no. (%) | | | | 0.806 |
| Yes | 20 (39) | 41 (39) | 16 (34) |  |
| No | 31 (61) | 63 (61) | 31 (66) |  |
| Baseline CEA level – no. (%) | | | | 0.054 |
| < 6 mg/dL | 34 (42) | 49 (30) | 32 (45) |  |
| ≥ 6 mg/dL | 47 (58) | 112 (70) | 39 (55) |  |
| Baseline CA199 level – no. (%) | | | | 0.130 |
| < 40 mg/dL | 48 (68) | 81 (53) | 38 (57) |  |
| ≥ 40 mg/dL | 23 (32) | 71 (47) | 29 (43) |  |

^a^ *P*-value by Fisher’s exact test

Abbreviations: EGFR, epidermal growth factor receptor; AJCC, American Joint Committee on Cancer**;** CEA, carcinoembryonic antigen**;** CA199, carbohydrate antigen 19-9

| **Supplemental Table S3**. **Baseline characteristics of responders and non-responders to anti-EGFR treatment among patients with middle/low rectal cancer** | | | | |
| --- | --- | --- | --- | --- |
| **Characteristic – no. (%)** | | **Responders**  **(*n* = 45)** | **Non-responders**  **(*n* = 101)** | ***P*-value** |
| Line of therapy | First-line | 31 | 41 |  |
|  | Non-first-line | 14 | 60 |  |
| Sex | Male | 29 (64) | 69 (68) | 0.646 |
|  | Female | 16 (37) | 32 (32) |  |
| Age | < 70 years | 37 (82) | 82 (81) | 0.882 |
|  | ≥ 70 years | 8 (18) | 19 (19) |  |
| AJCC stage | I to III | 14 (31) | 38 (38) | 0.448 |
|  | IV | 31 (69) | 63 (62) |  |
| Metastasectomy | Yes | 14 (31) | 34 (34) | 0.762 |
|  | No | 31 (69) | 67 (66) |  |
| Pathology | Adenocarcinoma | 44 (98) | 95 (94) | 0.332^a^ |
|  | Non-adenocarcinoma | 1 (2) | 6 (6) |  |
| Histological grade | High | 36 (90) | 76 (85) | 0.474 |
|  | Low | 4 (10) | 13 (15) |  |
| Mucinous component | Yes | 5 (15) | 19 (26) | 0.191^a^ |
|  | No | 29 (85) | 54 (74) |  |
| Signet cell component | Yes | 2 (6) | 3 (4) | 0.697^a^ |
|  | No | 32 (94) | 69 (96) |  |
| Lymphovascular invasion | Yes | 18 (53) | 35 (49) | 0.677 |
|  | No | 16 (47) | 37 (51) |  |
| Perineural invasion | Yes | 12 (48) | 24 (45) | 0.822 |
|  | No | 13 (52) | 49 (55) |  |
| Baseline CEA level | < 6 mg/dL | 18 (41) | 37 (41) | 0.978 |
|  | ≥ 6 mg/dL | 26 (59) | 54 (59) |  |
| Baseline CA199 level | < 40 mg/dL | 27 (66) | 54 (64) | 0.799 |
|  | ≥ 40 mg/dL | 14 (34) | 31 (36) |  |
| ^a^ *P*-value by Fisher’s exact test  Abbreviations: EGFR, epidermal growth factor receptor; AJCC, American Joint Committee on Cancer staging system**;** CEA, carcinoembryonic antigen**;** CA199, carbohydrate antigen 19-9 | | | | |

| \| **Supplemental Table S4. Progression-free survival and overall survival in patients with different lines of anti-EGFR treatment and primary tumour locations** \| \| \| \| \| \| --- \| --- \| --- \| --- \| --- \| \|  \| **First-line group** \| \|  \|  \| \|  \| **High/middle/low rectum^a^** \| **Left-sided colon** \| HR (95% CI) \| *P*-value \| \| PFS \|  \|  \|  \|  \| \| Median \| 8.0 (5.6–10.3) \| 9.3 (8.4–10.2) \| 0.80 (0.60–1.07) \| 0.136 \| \| OS \|  \|  \|  \|  \| \| Median \| 32.1 (25.1–39.1) \| 38.3 (30.7–45.9) \| 0.64 (0.45–0.90) \| 0.010 \| \|  \| **Non-first-line group** \| \|  \|  \| \|  \| **High/middle/low rectum^a^** \| **Left-sided colon** \| HR (95% CI) \| *P*-value \| \| PFS \|  \|  \|  \|  \| \| Median \| 4.4 (3.6–5.2) \| 5.6 (4.9–6.3) \| 0.87 (0.68–1.12) \| 0.289 \| \| OS \|  \|  \|  \|  \| \| Median \| 35.3 (30.3–40.3) \| 36.3 (31.0–41.6) \| 1.00 (0.76–1.32) \| 0.999 \|   ^a^ Rectal tumours are defined here as tumours located ≤ 15 cm from the anal verge.  Abbreviations: EGFR, epidermal growth factor receptor; PFS, progression-free survival**;** OS, overall survival; HR, hazard ratio; CI, confidence interval | | | | | | |
| --- | --- | --- | --- | --- | --- | --- | --- | --- | --- | --- | --- | --- | --- | --- | --- | --- | --- | --- | --- | --- | --- | --- | --- | --- | --- | --- | --- | --- | --- | --- | --- | --- | --- | --- | --- | --- | --- | --- | --- | --- | --- | --- | --- | --- | --- | --- | --- | --- | --- | --- | --- | --- | --- | --- | --- | --- | --- | --- | --- | --- | --- | --- | --- | --- | --- | --- | --- | --- | --- | --- | --- |
| **Supplemental Table S5. Tumour response in patients with different lines of anti-EGFR treatment and primary tumour locations** | | | | | | |
|  | **First-line group – *n* (%)** | | | **Non-first-line group – *n* (%)** | | |
|  | **High/middle/low rectum**^a^  **(*n*=94)** | **Left-sided colon**  **(*n*=122)** | *P*-value | **High/middle/low rectum**^a^  **(*n*=104)** | **Left-sided**  **colon**  **(*n*=164)** | *P*-value |
| Unevaluable patients | 8 | 14 |  | 20 | 18 |  |
| Complete response | 1 (1) | 4 (4) |  | 1 (1) | 2 (1) |  |
| Partial response | 41 (48) | 65 (60) |  | 15 (18) | 39 (27) |  |
| Stable disease | 35 (41) | 28 (26) |  | 41 (49) | 71 (49) |  |
| Progressive disease | 9 (10) | 11 (10) |  | 27 (32) | 34 (23) |  |
| Overall response rate | 42 (49) | 69 (64) | 0.035 | 16 (19) | 41 (28) | 0.127 |
| Disease control rate | 77 (90) | 97 (90) | 0.949 | 57 (68) | 112 (77) | 0.143 |

^a^ Rectal tumours are defined here as tumours located ≤ 15 cm from the anal verge.

Abbreviations: EGFR, epidermal growth factor receptor

**Supplemental Table S6. Univariate analysis of factors affecting progression-free survival and overall survival in the first-line anti-EGFR treatment group**

|  | **Univariate analysis** | | | | |
| --- | --- | --- | --- | --- | --- |
|  | **First-line PFS** | |  | **First-line OS** | |
| **Group** | **HR (95% CI)** | ***P*-value** |  | **HR (95% CI)** | ***P*-value** |
|  |  |  |  |  |  |
| Location (left vs mid/low rectum) | 0.67 (0.46–0.89) | 0.007 |  | 0.62 (0.44–0.88) | 0.008 |
| Sex (female) | 0.93 (0.69–1.26) | 0.646 |  | 1.00 (0.70–1.42) | 0.996 |
| Age (≥ 70 years) | 1.03 (0.73–1.45) | 0.873 |  | 1.78 (1.19–2.67) | 0.005 |
| AJCC stage | 0.94 (0.76–1.16) | 0.548 |  | 1.12 (0.87–1.46) | 0.381 |
| Metastasectomy | 0.56 (0.42–0.75) | <0.001 |  | 0.25 (0.17–0.36) | <0.001 |
| Pathology | 1.38 (0.86–2.21) | 0.178 |  | 1.11 (0.69–1.78) | 0.672 |
| Histological grade (high risk) | 1.69 (1.02–2.80) | 0.041 |  | 2.34 (1.32–4.24) | 0.004 |
| Mucinous component | 1.41 (0.90–2.23) | 0.137 |  | 0.99 (0.58–1.68) | 0.962 |
| Signet cell component | 1.13 (0.42–3.06) | 0.812 |  | 1.05 (0.33–3.32) | 0.940 |
| Lymphovascular invasion | 1.21 (0.87–1.69) | 0.253 |  | 1.29 (0.87–1.92) | 0.206 |
| Perineuronal invasion | 1.51 (1.03–2.20) | 0.034 |  | 1.55 (0.95–2.53) | 0.080 |
| CEA level (≥ 6 mg/dL) | 1.50 (1.06–2.12) | 0.022 |  | 1.48 (0.99–2.21) | 0.053 |
| CA199 level (≥ 40 mg/dL) | 1.29 (0.96–1.73) | 0.093 |  | 1.44 (1.01–2.04) | 0.044 |

Abbreviations: EGFR, epidermal growth factor receptor; PFS, progression-free survival**;** OS, overall survival; AJCC, American Joint Committee on Cancer staging system**;** CEA, carcinoembryonic antigen**;** CA199, carbohydrate antigen 19-9

**Supplemental Table S7. Univariate and multivariate analysis of factors affecting progression-free survival in the non-first-line anti-EGFR treatment group**

|  | **Non-first-line PFS** | | | | |
| --- | --- | --- | --- | --- | --- |
|  | **Univariate analysis** | |  | **Multivariate analysis** | |
| **Group** | **HR (95% CI)** | ***P*-value** |  | **HR (95% CI)** | ***P*-value** |
|  |  |  |  |  |  |
| Location (left vs mid/low rectum) | 0.73 (0.56–0.95) | 0.019 |  | 0.67 (0.50–0.91) | 0.009 |
| Sex (female) | 1.20 (0.93–1.55) | 0.166 |  | 1.22 (0.93–1.61) | 0.147 |
| Age (≥ 70 years) | 0.83 (0.62–1.12) | 0.229 |  | 0.88 (0.64–1.21) | 0.432 |
| AJCC stage | 0.97 (0.81–1.15) | 0.701 |  | 1.00 (0.80–1.24) | 0.990 |
| Metastasectomy | 0.65 (0.50–0.84) | 0.001 |  | 0.62 (0.47–0.82) | 0.001 |
| Pathology | 1.18 (0.86–1.61) | 0.300 |  | 0.74 (0.45–1.22) | 0.236 |
| Histological grade (high risk) | 1.91 (1.04–3.52) | 0.037 |  | 1.35 (0.61–3.00) | 0.466 |
| Mucinous component | 1.55 (1.11–2.18) | 0.011 |  | 1.80 (1.22–2.67) | 0.003 |
| Signet cell component | 1.63 (0.72–3.69) | 0.238 |  | 0.96 (0.24–3.84) | 0.952 |
| Lymphovascular invasion | 1.34 (1.01–1.78) | 0.042 |  | 1.51 (1.03–2.22) | 0.036 |
| Perineuronal invasion | 1.51 (1.08–2.12) | 0.016 |  | 1.34 (0.92–1.96) | 0.127 |
| CEA level (≥ 6 mg/dL) | 1.00 (0.76–1.32) | 0.981 |  | 1.15 (0.81–1.63) | 0.444 |
| CA199 level (≥ 40 mg/dL) | 0.92 (0.70–1.21) | 0.557 |  | 0.72 (0.52–0.98) | 0.039 |

Abbreviations: EGFR, epidermal growth factor receptor; PFS, progression-free survival; AJCC, American Joint Committee on Cancer staging system**;** CEA, carcinoembryonic antigen**;** CA199, carbohydrate antigen 19-9

**Supplemental Table S8. Univariate and multivariate analysis of factors affecting overall survival in the non-first-line anti-EGFR treatment group**

|  | **Non-first-line OS** | | | | |
| --- | --- | --- | --- | --- | --- |
|  | **Univariate analysis** | |  | **Multivariate analysis** | |
| **Group** | **HR (95% CI)** | ***P*-value** |  | **HR (95% CI)** | ***P*-value** |
|  |  |  |  |  |  |
| Location (left vs mid/low rectum) | 0.90 (0.68–1.20) | 0.479 |  | 1.06 (0.77–1.47) | 0.729 |
| Sex (female) | 1.60 (1.20–2.12) | 0.001 |  | 1.97 (1.45–2.68) | <0.001 |
| Age (≥ 70 years) | 1.11 (0.80–1.55) | 0.530 |  | 0.95 (0.66–1.36) | 0.767 |
| AJCC stage | 1.18 (0.97–1.44) | 0.098 |  | 1.04 (0.81–1.33) | 0.767 |
| Metastasectomy | 0.40 (0.30–0.54) | <0.001 |  | 0.40 (0.29–0.55) | <0.001 |
| Pathology | 0.86 (0.60–1.21) | 0.328 |  | 0.52 (0.30–0.89) | 0.017 |
| Histological grade (high risk) | 1.64 (0.86–3.10) | 0.131 |  | 1.08 (0.45–2.61) | 0.859 |
| Mucinous component | 1.27 (0.88–1.83) | 0.202 |  | 1.65 (1.07–2.54) | 0.023 |
| Signet cell component | 1.59 (0.65–3.89) | 0.309 |  | 4.22 (0.94–19.0) | 0.061 |
| Lymphovascular invasion | 1.70 (1.24–2.34) | 0.001 |  | 2.17 (1.40–3.35) | 0.001 |
| Perineuronal invasion | 1.39 (0.95–2.02) | 0.088 |  | 0.99 (0.67–1.48) | 0.972 |
| CEA level (≥ 6 mg/dL) | 1.81 (1.33–2.47) | <0.001 |  | 1.98 (1.36–2.88) | <0.001 |
| CA199 level (≥ 40 mg/dL) | 1.42 (1.05–1.93) | 0.025 |  | 0.86 (0.60–1.23) | 0.401 |

Abbreviations: EGFR, epidermal growth factor receptor; OS, overall survival; AJCC, American Joint Committee on Cancer staging system**;** CEA, carcinoembryonic antigen**;** CA199, carbohydrate antigen 19-9

| **Supplemental Table S9. Baseline characteristics of the patients who underwent metastasectomy among first-line anti-EGFR treatment recipients with different primary tumour locations** | | | | |
| --- | --- | --- | --- | --- |
| **Characteristic – no. (%)** | **Middle/low rectum (*n* = 25)** | **Left-sided colon  (*n* = 67)** | **Right-sided colon (*n* = 10)** | ***P*-value** |
| Sex – no. (%) | | | | 0.251^b^ |
| Male | 17 (68) | 45 (67) | 4 (40) |  |
| Female | 8 (32) | 22 (33) | 6 (60) |  |
| Age – no. (%) | | | | 0.017^b^ |
| < 70 years | 23 (92) | 55 (82) | 4 (40) |  |
| ≥ 70 years | 2 (8) | 12 (18) | 6 (60) |  |
| AJCC stage – no. (%) | | | | 0.007^b^ |
| I to III | 10 (40) | 9 (13) | 0 (0) |  |
| IV | 15 (60) | 58 (87) | 10 (100) |  |
| Induction therapy^a^ – no. (%) | | | | 0.332^b^ |
| Yes | 16 (64) | 44 (66) | 4 (40) |  |
| No | 9 (36) | 23 (34) | 6 (60) |  |
| Surgery sequence – no. (%) | | | | 0.462^b^ |
| Combined resection | 12 (48) | 32 (48) | 3 (30) |  |
| Sequential resection | 13 (52) | 35 (52) | 7 (70) |  |
| Metastatic sites – no. (%) | | | | 0.053^b^ |
| Liver-limited metastasis | 11 (44) | 43 (64) | 4 (40) |  |
| Lung-limited metastasis | 5 (20) | 5 (7) | 3 (30) |  |
| Other single-site metastasis | 3 (12) | 1 (1) | 0 (0) |  |
| > 1 metastatic site | 6 (24) | 18 (27) | 3 (30) |  |
| ^a^ Anti-EGFR treatment as induction therapy.  ^b^ *P*-value by Fisher’s exact test.  Abbreviations: EGFR, epidermal growth factor receptor**;** AJCC, American Joint Committee on Cancer staging system | | | | |

| **Supplemental Table S10. Baseline characteristics of the patients who underwent colorectal cancer hepatic metastasectomy among first-line anti-EGFR treatment recipients with different primary tumour locations** | | | | |
| --- | --- | --- | --- | --- |
| **Characteristic – no. (%)** | **Middle/low rectum (*n* = 14)** | **Left-sided colon  (*n* = 56)** | **Right-sided colon (*n* = 7)** | ***P*-value** |
| Number of metastasis – no. (%) | | | | 0.194^a^ |
| ≤ 3 | 12 (86) | 35 (63) | 6 (86) |  |
| > 3 | 2 (14) | 21 (38) | 1 (14) |  |
| Maximal size of metastasis – no. (%) | | | | 0.243^a^ |
| ≤ 3 cm | 9 (64) | 29 (52) | 6 (86) |  |
| > 3 cm | 5 (36) | 27 (48) | 1 (14) |  |
| Metastasis distribution – no. (%) | | | | 0.447^a^ |
| Unilobar | 12 (86) | 38 (68) | 5 (71) |  |
| Bilobar | 2 (14) | 18 (32) | 2 (29) |  |
| Resection margin – no. (%) | | | | 0.413^a^ |
| R0 resection | 14 (100) | 49 (88) | 7 (100) |  |
| R1 resection | 0 (0) | 7 (13) | 0 (0) |  |
| ^a^ *P*-value by Fisher’s exact test  Abbreviations: EGFR, epidermal growth factor receptor | | | | |
